# Biological threat preparedness through vaccine development and stockpiling: challenges and strategic implications

**DOI:** 10.3389/fpubh.2025.1614626

**Published:** 2025-06-02

**Authors:** Jungeun Lee

**Affiliations:** Guided and Firepower Systems Technology Planning Team, Korea Research Institute for Defense Technology Planning and Advancement, Jinju, Republic of Korea

**Keywords:** biological threats, vaccine stockpiling, vaccine platform technologies, weaponizable biological agents, biodefense strategy, public health preparedness

## Abstract

Biological threat agents such as *Bacillus anthracis*, *Variola virus*, and botulinum toxin pose serious risk to national security and public health due to their high transmissibility, lethality, and potential for weaponization. This study analyzes the current status of vaccine development and strategic stockpiling for five biological agents—*B. anthracis*, *Variola virus*, *Yersinia pestis*, *Vibrio cholerae*, and botulinum toxin—which are believed to be potentially weaponized by North Korea. It reviews both traditional and next-generation vaccine platforms, including live-attenuated, inactivated, protein subunit, viral vector, DNA, RNA, and novel technologies such as self-amplifying RNA vaccine and advanced adjuvants. The study also examines the vaccine stockpiling strategies of major countries and international organizations, with a focus on key pathogens, logistical frameworks, and policy implications. Based on the findings, the paper highlights the need for enhanced global cooperation, public–private partnerships, and long-term investment to improve vaccine preparedness. Developing rapid deployment systems under military coordination, along with harmonizing international vaccine-sharing protocols, is considered essential for strengthening biodefense and emergency response readiness.

## 1 Introduction

On May 28, 2024, North Korea deployed numerous balloons carrying waste materials into South Korean airspace. Although initially perceived as a form of psychological warfare, the discovery of organic matter—including feces and manure—sparked concern that these objects may have served as an experimental vehicle for dispersing biological agents. The event reignited long-standing concerns about North Korea’s suspected biological weapons program, which continues to pose serious risks to national security and public health. In response, members of the South Korean parliament cited the potential for deployment of *Bacillus anthracis* (anthrax) and *Variola virus* (smallpox) by North Korea, stressing the critical need for strategic vaccine stockpiles. While some experts have questioned the technical feasibility of balloon-based delivery, the broader implications for regional security remain unresolved (1).

At the same time, the global health system remains fragile in the aftermath of the COVID-19 pandemic. While the World Health Organization (WHO) declared an end to the Public Health Emergency of International Concern (PHEIC) in May 2023, SARS-CoV-2 has become endemic, with new variants still emerging. Meanwhile, the return of diseases like monkeypox and cholera continues to highlight ongoing weaknesses in global preparedness and response systems.

Despite being a party to the Biological Weapons Convention (BWC), North Korea has frequently been cited by South Korea’s Defense White Papers and U.S. State Department reports as a suspected possessor of biological weapons (2, 3). While the U. S. Arms Control Compliance Report does not name specific agents, South Korean defense and security analyses have identified five biological agents—*B. anthracis*, *Variola virus*, *Yersinia pestis*, *Vibrio cholerae*, and botulinum toxin—as the most likely to be weaponized by North Korea, based on their lethality, transmission potential, and feasibility of production (4). These same agents are classified as top-tier biological threats by both the U. S. Centers for Disease Control and Prevention (CDC) and WHO.

Given the continued threat posed by biological agents, maintaining strategic vaccine reserves remains a key pillar of biodefense, not only for mitigating biological attack scenarios, but also for deterring adversaries from employing high-impact biological agents such as *B. anthracis* or *Variola virus* by reducing their operational value. This review provides a detailed analysis of vaccine development and preparedness strategies focused on five agents considered among the most likely to be weaponized by North Korea. It contrasts traditional and next-generation vaccine platforms, evaluates current national and international stockpiling systems, and identifies critical policy areas that require strengthening. By connecting the biological features of these pathogens with their operational relevance and gaps in global readiness, the review aims to support the formulation of integrated bioterrorism response strategies at both national and international levels.

While this review primarily examines vaccine development and stockpiling from biodefense perspective, it adopts a strategic focus on the role of vaccines as military countermeasures against biological weapons. In particular, it addresses scenarios where preemptive vaccination may be warranted for military personnel operating in high-risk biological threat environments. Although real-time vaccination in response to a biological attack presents logistical and immunological limitations, stockpiling remains essential when facing biological agents that are highly contagious—such as *Variola virus*—or environmentally persistent—such as *B. anthracis*. In such cases, vaccines may function not only as protective tools but also as deterrents by discouraging adversaries from employing the most devastating biological agents.

At the same time, the insights drawn from this analysis remain relevant to public health preparedness more broadly. The COVID-19 pandemic has demonstrated that vaccine development speed, supply chain resilience, and deployment infrastructure are just as critical in responding to naturally emerging threats. Thus, while this study emphasizes military readiness, the strategic frameworks discussed here may also contribute to building more resilient public systems.

## 2 Biological threat landscape

North Korea’s suspected biological weapons program poses a persistent threat to regional and global security. While the regime remains a signatory of the BWC, numerous defense white papers and intelligence assessments from South Korea and the United States have consistently raised concerns over the nation’s clandestine development and potential deployment of biological agents. Recent incidents, such as the release of balloon-borne waste materials into South Korea in 2024, have reignited speculation about experimental delivery systems for biological warfare.

Among the biological agents suspected to be stockpiled by North Korea, five—*B. anthracis*, *Variola virus*, *Y. pestis*, *V. cholerae*, and botulinum toxin—stand out due to their extreme lethality, high transmissibility, environmental resilience and persistence, and relative ease of weaponization. These agents are also classified as Tier 1 threats by both the CDC and the WHO, highlighting their potential to inflict large-scale casualties and disrupt critical societal functions.

Each of these pathogens exhibits distinct virulence characteristics that complicate early detection, containment, and treatment efforts. *B. anthracis* produces spores capable of remaining viable in the environment for decades, while *Variola virus*, the cause of smallpox, was historically responsible for substantial mortality, and precisely because it has been globally eradicated and routine vaccination has ceased, it now poses an exceptionally potent biological threat. *Y. pestis* and *V. cholerae*—which cause plague and cholera—remain acute public health threats due to their ability to spread rapidly under favorable conditions. Botulinum toxin, among the most potent biological substances known, can lead to life-threatening paralysis even at extremely low exposure levels. However, historical evaluations have noted that despite its extreme toxicity, botulinum toxin had limited effectiveness as aerosol weapon due to environmental and delivery constraints (5).

Understanding the properties and risks associated with these agents is essential for guiding vaccine R&D priorities and informing national and international biodefense policies. The following sections provide an overview of current vaccine technology platforms and review countermeasure development efforts tailored to each of these high-concern agents.

## 3 Vaccine platform technologies

The growing concern over biological threats has spurred meaningful advancements in vaccine development technologies. Central to this progress are vaccine platforms—the technological foundations that enable the generation of protective immune responses. Traditional approaches, such as live-attenuated and inactivated vaccines, have long served as the backbone of global immunization programs. In addition, the refinement of recombinant protein expression techniques has led to broader adoption of protein subunit vaccines.

More recently, innovative platforms like viral vectors, nucleic acid–based vaccines (DNA and RNA), and virus-like particles (VLPs) have emerged as promising tools for rapid and scalable production. Alongside these developments, progress in adjuvant design has significantly enhanced vaccine immunogenicity, expanding the range of effective options for biodefense applications (6).

### 3.1 Traditional vaccine platforms

Traditional vaccine platforms—including live-attenuated, inactivated, and protein subunit vaccines—have served as the cornerstone of global disease prevention efforts.

Live-attenuated vaccines are formulated from pathogens that have been weakened to minimize virulence while maintaining replication competency in the host. These vaccines typically induce robust and long-lasting immune responses that mimic natural infection. However, booster administration may still be required, depending on the vaccine. Importantly, live-attenuated vaccines are generally contraindicated in immunocompromised individuals due to the low but present risk for pathogenesis and potential for reversion to a virulent phenotype. Representative examples include the smallpox vaccine derived from *Vaccinia* virus and the attenuated *Y. pestis* vaccine developed in the former Soviet Union.

Inactivated vaccines are prepared by chemically or physically killing the pathogen, thereby eliminating its ability to replicate while preserving key immunogenic components. These vaccines are considered relatively safe due to the absence of live agents but generally induce weaker immune responses. As a result, they are often formulated with adjuvants and may require multiple boosters. A representative example is BioThrax®, an inactivated vaccine developed for protection against *B. anthracis*.

Protein subunit vaccines are produced using recombinant technology to express specific antigens from target pathogens. These purified proteins are then administered to stimulate an immune response. While this platform offers excellent safety profiles, it generally exhibits lower immunogenicity than other vaccine types, in some cases, often requiring the addition of adjuvants to improve efficacy. An example is NVX-CoV2373, developed by Novavax during the COVID-19 pandemic.

While conventional vaccine platforms remain fundamental in infectious disease control, their application to biodefense scenarios presents notable constraints. Developing vaccines against high-risk biological agents typically requires biosafety level 3 (BSL-3) or higher containment, which limits both production speed and scalability. These logistical hurdles have contributed to an increasing reliance on next-generation vaccine technologies that provide improved flexibility and significantly reduced development timelines.

### 3.2 Next-generation vaccine platforms

Next-generation vaccine platforms have emerged as a direct response to the growing need for flexible and fast vaccine development. Among the most notable advances are viral vector, DNA, RNA, and VLP vaccines—technologies that received accelerated attention during the COVID-19 pandemic (7).

Viral vector-based vaccines involve the use of genetically modified, non-replicating viruses to transport antigen-encoding genetic material into host cells. This approach triggers strong and durable immune responses and offers the advantage of rapid redesign to combat newly emerging variants by altering the vector’s genetic code. Nevertheless, pre-existing immunity to the viral vector, or the development of anti-vector immune responses after administration, can limit overall effectiveness. There is also a risk that spontaneous mutations within the vector could impair its immunogenic properties. Prominent examples include Jcovden (JNJ-78436735) by Johnson & Johnson and Vaxzevria (AZD1222) by AstraZeneca, both deployed during the COVID-19 crisis.

DNA vaccines operate by introducing plasmid DNA encoding target antigens into the host, where the genes are transcribed and translated into immunogenic proteins. These vaccines are thermally stable, easy to store, and suitable for large-scale manufacturing. Nevertheless, their inherently low immunogenicity often requires the use of adjuvants or advanced delivery technologies to enhance immune activation. A representative product is ZyCoV-D, created by Cadila Healthcare in India.

RNA vaccines work by introducing messenger RNA (mRNA) encased in lipid nanoparticles (LNPs), which help deliver genetic instructions into the host’s cells. Once delivered, the cellular machinery uses the mRNA to produce specific antigens, triggering an immune response targeted at the pathogen. This method has shown high levels of immunogenicity and can be rapidly adapted to combat emerging infectious diseases. The success of vaccines like Comirnaty (BNT162b2, Pfizer–BioNTech) and Spikevax (mRNA-1273, Moderna) during the COVID-19 pandemic demonstrates the transformative potential of this technology. However, one major challenge lies in the inherent instability of mRNA, which complicates storage and transport. While mRNA is generally considered to degrade rapidly *in vivo*, recent studies have reported case of prolonged persistence, highlighting the importance of analyte stability and precise target selection in mRNA vaccine design (8).

VLP vaccines replicate the external structure of actual viruses by utilizing surface antigen proteins, but lack any genetic material, thereby eliminating the possibility of infection. Compared to conventional protein subunit vaccines, VLPs tend to induce stronger immune responses and are associated with enhanced safety profiles. As a result, they are regarded as a promising platform for next-generation vaccine development. Several VLP-based vaccines have already received regulatory approval, including those targeting hepatitis B, human papillomavirus (HPV), and malaria.

Next,-generation vaccine platforms offer clear advantages over traditional methods, especially in biodefense scenarios. Still, each platform differs significantly in areas like development cost, durability of immune responses, ease of production, and storage logistics. Because of these differences, choosing the right platform for a specific biological threat demands a careful evaluation of both the pathogen’s unique traits and the real-world conditions under which the vaccine must be deployed. Designing vaccine strategies with these factors in mind is crucial to sustainment of the program ensuring a timely and effective response.

**Figure 1:**
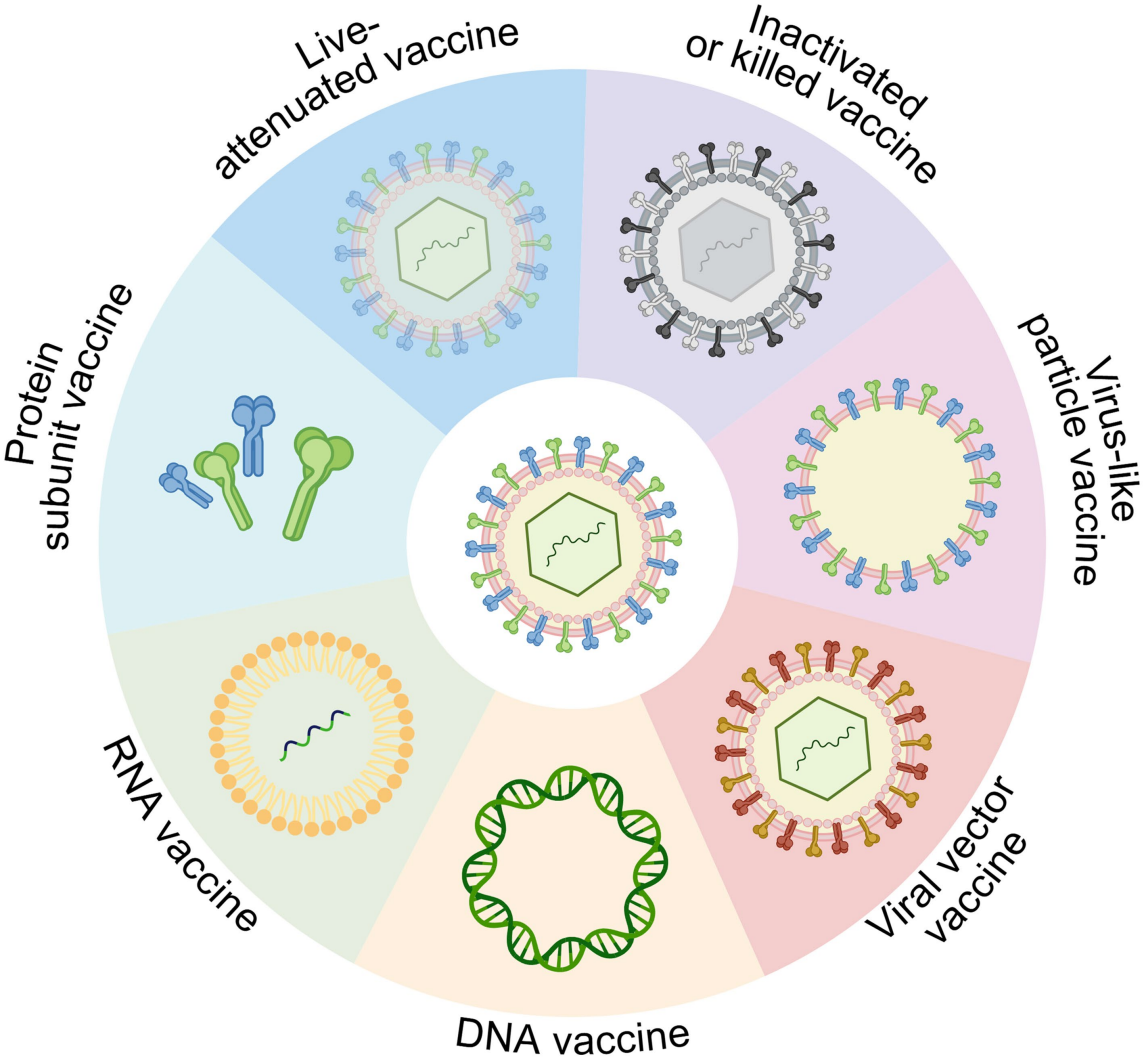
Overview of vaccine platform technologies, including live-attenuated, inactivated (killed), protein subunit, viral vector, DNA, RNA, and virus-like particle (VLP) vaccines. Each platform differs in antigen delivery mechanism, immunogenicity, and applicability for rapid development and biodefense use. Created in BioRender. https://BioRender.com/14m8zdd.

In the field of vaccine development, platform technologies differ widely in immunogenicity, safety, scalability, and stability. Table 1 provides a general overview of the advantages and limitations of each vaccine platform, which may inform decisions in both public health and biodefense contexts. To visually complement this overview, Figure 1 presents a structural diagram of the major vaccine platform types.

**Table 1:** General advantages and disadvantages of vaccine platforms relevant to biodefense and public health.

Platform	Advantages	Disadvantages
Live-attenuated	Robust and durable immune response Effective at inducing both humoral and cellular immunity	Safety concerns in immunocompromised individuals Complex manufacturing and cold chain requirements
Inactivated	High safety profile Ease of storage and transport	Generally weaker immune response May require booster doses
Protein subunit	Excellent safety profile Targeted immune response possible	Lower immunogenicity; requires adjuvants Complex purification and formulation process
Viral vector	High immunogenicity Efficient intracellular delivery	Pre-existing immunity to the vector may reduce efficacy Risk of vector-specific immune responses
DNA	Thermostability and rapid design Capable of inducing both arms of adaptive immunity	Requires entry into the nucleus Limited immunogenicity in humans
mRNA	Rapid and scalable production Capable of inducing both arms of adaptive immunity Can generate strong immune responses	Requires ultra-cold storage Stability issues with RNA and lipid nanoparticles (LNPs) Requires regular boosters due to limited long-term immunity Concerns regarding cardiac side effects in young individuals, particularly athletes (80)

### 3.3 Emerging technologies in vaccine development

In addition to conventional vaccine platforms, recent breakthroughs—such as viral vector, DNA, and mRNA vaccines—have significantly accelerated vaccine development, particularly during the COVID-19 pandemic. However, important questions remain regarding their long-term efficacy, durability of immune protection, and the clearance kinetics of mRNA within the body. Even so, the experience from this period revealed several critical shortcomings—from safety concerns and cold-chain limitations to brief antigen expression—that highlight the need for ongoing innovation. To address these challenges, researchers have begun exploring new solutions, including self-amplifying RNA (saRNA) and circular RNA (circRNA) vaccines, as well as next-generation adjuvant technologies designed to enhance both the intensity and longevity of immune responses.

saRNA vaccines represent an advanced platform designed to overcome some of the limitations of conventional mRNA vaccines. Unlike standard mRNA vaccines, saRNA includes not only the antigen-encoding sequence but also viral replicase genes, enabling the RNA to self-replicate inside host cells (9). This facilitates strong and sustained antigen expression with lower doses. A notable example is ARCT-154, developed by Arcturus Therapeutics in Japan, which became the world’s first saRNA vaccine to be approved, in November 2023, for COVID-19. BNT162c2 by BioNTech is another candidate currently undergoing clinical evaluation. Beyond COVID-19, saRNA vaccines are also being explored for use against influenza, Ebola, HIV-1, and other infectious diseases (10).

Unlike conventional linear mRNA platforms, circRNA vaccines feature a covalently closed-loop structure that makes them more resistant to RNase degradation, enabling longer-lasting antigen expression *in vivo* (11). Preclinical studies in mice and non-human primates have demonstrated that circRNA vaccines offer extended antigen production and stronger immune responses compared to their linear counterparts, suggesting they could play an important role in tackling future infectious disease threats (12).

At the same time, advances in adjuvant technologies are gaining attention for their ability to improve vaccine performance. Adjuvants work by enhancing immune responses and supporting more effective antigen presentation. However, traditional adjuvants—like aluminum salts or water-in-oil emulsions—often fall short in triggering robust cellular immunity and tend to be less effective in older individuals. Moreover, the exact mechanisms behind some of these agents remain unclear, raising concerns about their safety if not carefully controlled. To address these limitations, researchers are investigating a new generation of adjuvants, including synthetic double-stranded RNA, metabolic enhancers, manganese compounds, and nanoparticle-based delivery systems—all of which have shown encouraging results in early-stage studies.

In short, next-generation vaccine technologies are helping to overcome the constraints of older approaches by enabling quicker, stronger, and longer-lasting immunity. Continued progress in genetic vaccine design, adjuvant development, and precision delivery will likely play a major role in future preparedness—whether for existing diseases that do not yet have an effective vaccine, emerging diseases or engineered biological threats.

## 4 Trends in vaccine development for high-risk biological agents

North Korea is reportedly continuing to expand its capabilities in the development and potential deployment of biological weapons, according to open-source assessments and expert analyses (13). Among the agents suspected to be part of its arsenal, five pathogens—*B. anthracis*, *Variola virus*, *Y. pestis*, *V. cholerae*, and botulinum toxin—are regarded as having particularly high potential for weaponization, although historical assessments have questioned the effectiveness of botulinum toxin as an aerosolized weapon due to environmental and delivery limitations (5). These biological agents are characterized by high lethality and transmissibility. Among them, *B. anthracis* is notable for its environmental persistence due to the durability of its spores, while the others generally degrade more rapidly in the environment unless protected through specialized means. These properties contribute to their consideration as potential candidates for use in weapons of mass destruction.

Should North Korea choose to deploy biological weapons in actual combat, it is assessed to possess multiple delivery capabilities, including munitions, sprayers, and aerosol dissemination devices (4). The first method is aerial dissemination using aircraft and helicopters. North Korea operates IL-28 and MiG-series fighters, AN-2 light aircraft, and helicopters such as the Mi-2, Mi-4, and Mi-8, which could be utilized to disperse biological agents across wide areas if equipped with appropriate spray tanks or munitions specifically designed for biological delivery. Although this method can contaminate large regions, it is susceptible to detection and weather conditions.

The second delivery method involves using missile and artillery systems to disperse biological agents. North Korea’s sizable arsenal—including FROG and SCUD missiles, as well as a range of long-range artillery—could allow for the deployment of these agents over large distances. Equipping such weapons with biological warheads would enable both targeted strikes and broad-area contamination, significantly increasing the threat level. However, effective dissemination of viable biological agents via missile delivery would require specialized warhead design, including thermal protection and mechanisms such as submunitions to ensure proper aerosolization upon release.

The third approach leverages biological vectors such as infected animals or insects. Carriers like rodents, lice, fleas, and mosquitoes could be used to spread pathogens indirectly, leading to outbreaks that may be difficult to detect and easy to misinterpret as naturally occurring epidemics.

The fourth tactic relies on covert operations carried out by specially trained military units. North Korea is believed to maintain elite forces capable of infiltrating target areas and discreetly releasing pathogens or contaminated substances using concealed dispersal devices. Because of the stealth involved, these attacks could trigger localized outbreaks without warning, representing a highly asymmetric and unconventional form of biological warfare.

Given North Korea’s assessed capability to deliver biological agents through diverse means, the importance of vaccine development as a countermeasure is increasingly underscored. The five biological agents mentioned above are categorized as having a high potential for bioweaponization due to their lethality and ease of spread.

Table 2 summarizes the key characteristics of the five biological agents suspected to be weaponized by North Korea, including their transmission routes, clinical manifestations, incubation periods, and case fatality rates. Based on this overview, the following sections provide a detailed review of vaccine development trends and the current research status for each pathogen.

**Table 2:** Summary of key characteristics of five biological agents potentially weaponized by North Korea.

Category	Agent (pathogen)	Infection route	Latent period	Symptoms	Lethality
Bacteria	*Bacillus anthracis*	Inhalation, cutaneous exposure, ingestion	1–6 days	Pneumonia, septicemia	>90%
	*Yersinia pestis*	Inhalation, rat flea	2–4 days	Hemoptysis, chills, severe fever	90–100%
	*Vibrio cholerae*	Ingestion or consumption of contaminated water	1–5 days	Diarrhea, dehydration, hypotension	50% (without treatment)
Virus	*Variola virus*	Inhalation, cutaneous exposure or contact exposure	7–9 days	Mucosal bleeding, skin blistering, severe fever	20–40%
Toxin	Botulinum toxin	Inhalation, ingestion, cutaneous exposure	1–4 days	Headache, dizziness, thirst, mydriasis, flaccid paralysis	65% (e.g., depending on exposure type)

### 4.1 Anthrax vaccines

**Figure 2:**
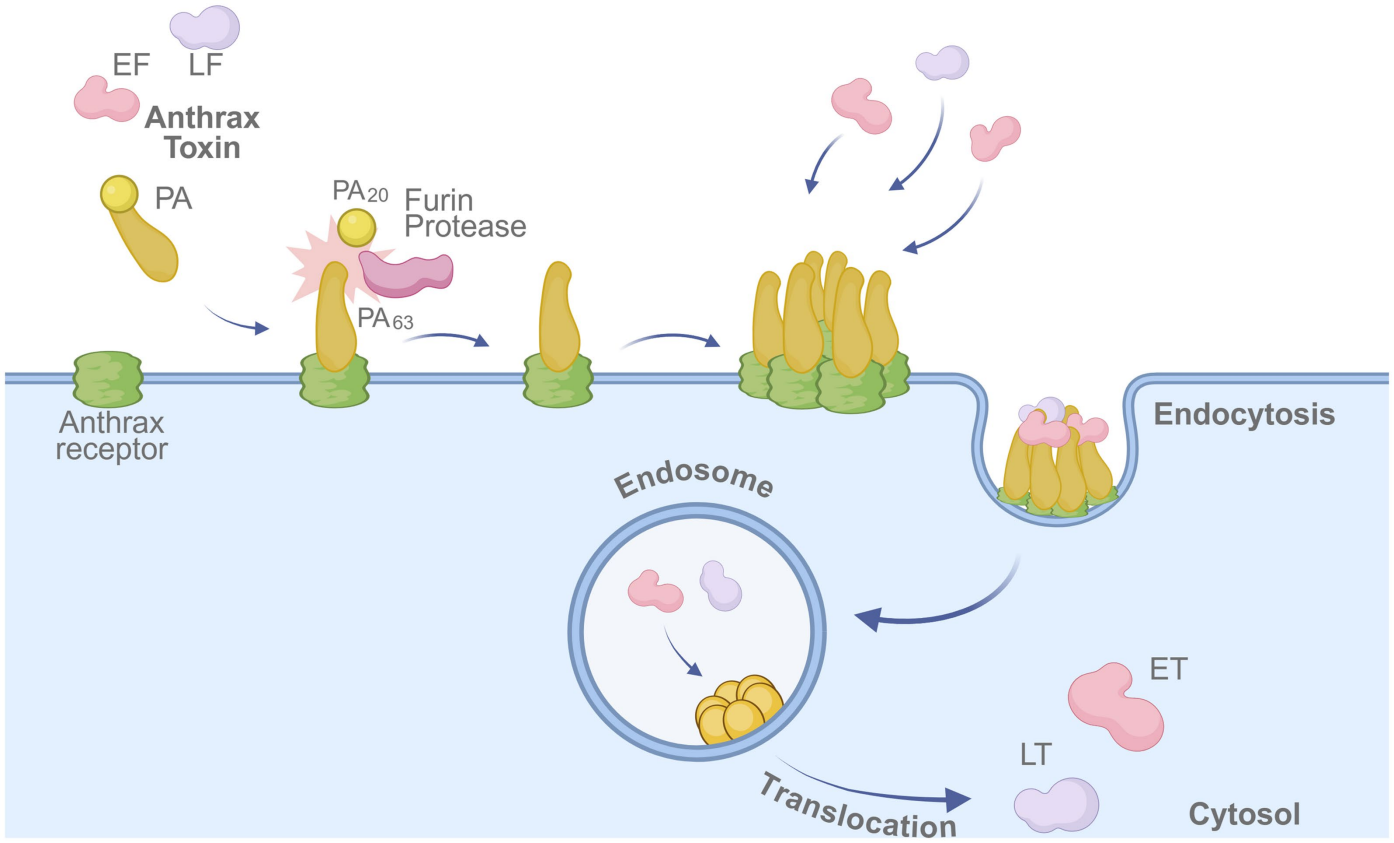
Mechanism of anthrax toxin entry into host cells. The protective antigen (PA) binds to the anthrax receptor on the host cell membrane and is cleaved by furin protease into PA63. The cleaved PA oligomerizes, enabling binding of lethal factor (LF) and edema factor (EF). The toxin complex is internalized via endocytosis, and LF/EF are translocated into the cytosol, where they exert cytotoxic effects. Created in BioRender. https://BioRender.com/fu6awpz.

Anthrax is a zoonotic infectious disease caused by *B. anthracis*. Infection can occur through inhalation, ingestion, or contact with skin lesions. *B. anthracis* is a spore-forming bacterium, which allows it to survive in the environment for extended periods and can cause fatal disease in both humans and animals. Due to its high lethality and resilience, it is considered one of the pathogens with the greatest potential for use as a biological weapon. The bacterium produces protective antigen (PA), lethal factor (LF), and edema factor (EF); PA combines with LF and EF to form lethal toxin (LT) and edema toxin (ET). Most anthrax vaccines target PA as the main immunogenic component and have been developed to provide protection and prevent disease manifestation. The entry mechanism of anthrax toxin into host cells underscores the central role of PA in vaccine design (Figure 2).

In the United States, two anthrax vaccines have been approved by the Food and Drug Administration (FDA): BioThrax^®^ (Anthrax Vaccine Adsorbed, AVA) developed by Emergent BioSolutions, and CYFENDUS^™^ (AV7909). BioThrax^®^ is produced from a culture filtrate of a non-virulent *B. anthracis* strain (V770-NP1-R) (14). Approved in 2015, the vaccine has been administered in more than 8.7 million doses to over 2.2 million U. S. military personnel since 1998. CYFENDUS™ is a next-generation anthrax vaccine that combines BioThrax® with CPG 7909 adjuvant to enhance the immune response. It is approved for post-exposure prophylaxis in individuals with confirmed or suspected exposure to *B. anthracis* to prevent disease manifestation.

In the United Kingdom, Anthrax Vaccine Precipitated (AVP), developed by Porton Biopharma, has been approved and is produced using a culture filtrate of the non-virulent *B. anthracis* Sterne strain (14). In Russia, the Kirov Institute of Microbiology developed a live-attenuated anthrax vaccine (LAAV) using the STI-1 strain (15), while China has also developed a live vaccine using the A16R attenuated strain of *B. anthracis*.

Concerns regarding the need for booster vaccinations and adverse effects of existing anthrax vaccines have driven research into second-generation recombinant PA (rPA)-based vaccines. Multiple vaccine candidates are currently being evaluated for safety and efficacy through clinical trials. For instance, DynPort Vaccine Company completed a Phase 1 trial of an *Escherichia coli*-derived rPA vaccine (NCT00057525). VaxGen developed rPA102 and completed Phase 1 and 2 trials (NCT00103467, NCT00100724), after which the technology was acquired by Emergent BioSolutions. PharmAthene’s rPA vaccine, SparVax^®^, also completed Phase 2 trials (NCT00170456, NCT00170469).

In South Korea, GC Biopharma (formerly Green Cross) has developed GC1109, an rPA-based anthrax vaccine that has completed Phase 1 and 2 clinical trials and is currently under regulatory review by the Ministry of Food and Drug Safety (MFDS) (NCT01867957, NCT01624532) (16). In Germany, the Fraunhofer Center for Molecular Biotechnology developed PA83-FhCBM, a plant-derived rPA vaccine, which completed a Phase 1 trial (NCT02239172) (17). Pfenex also completed a Phase 1 trial of Px563L (with adjuvant and RPA563) and RPA563, both based on mutant recombinant PA (NCT02655549) (18). Research on intranasal rPA vaccines is ongoing as well. BlueWillow developed BW-1010, an intranasal vaccine using the NanoVax system with Porton Biopharma’s rPA and oil-in-water nanoemulsion adjuvant, and completed a Phase 1 trial (NCT04148118). Given its delivery route, this vaccine may provide enhanced mucosal immunity and could offer improved protection against aerosolized anthrax spores compared to injectable formulations.

Viral vector vaccine candidates have also been developed. PaxVax, in collaboration with Emergent BioSolutions, completed a Phase 1 trial for adenovirus type 4-based Ad4-PA and Ad4-PA-GPI (glycosylphosphatidylinositol) vaccines (NCT01979406). Altimmune developed an adenovirus-based vaccine, Nanoshield, which completed a Phase 1b trial (NCT03352466). In addition, an adenovirus type 5-based intranasal vaccine (Ad-AVA) demonstrated protective effects in both mouse and rabbit models (19, 20).

DNA vaccine research is also actively progressing. DNA vaccines that express PAD4 (protective antigen domain 4) or co-express PA and *B. anthracis* surface protein EA1 (extractable antigen 1) have shown protective efficacy in mice through intradermal and intraperitoneal administration, respectively (21, 22). A dual-expression system-based multipathogen DNA vaccine, which encodes PAD4 of *B. anthracis* and HCt (C-terminal fragment of the heavy chain) of *Clostridium botulinum*, has also demonstrated protection in murine models against both *B. anthracis* and botulinum toxin (23).

Table 3 summarizes key anthrax vaccines currently in use or under development, focusing on their platforms, indications, and regulatory status. These include traditional live-attenuated formulations as well as modern recombinant protein-based vaccines targeting PA of *B. anthracis*.

**Table 3:** Major anthrax vaccines currently in development and use.

Vaccine name	Developer/country	Platform	Indication	Development stage/approval
BioThrax^®^ (AVA)	Emergent BioSolutions/United States	Cell-free filtrate (V770-NP1-R)	Pre-exposure prophylaxis (military use)	FDA approved; widely used since 1998
CYFENDUS^™^ (AV7909)	Emergent BioSolutions/United States	rPA + CPG 7909 adjuvant	Post-exposure prophylaxis	FDA approved (2023)
AVP	Porton Biopharma/UK	Live-attenuated (Sterne 34F2)	Pre-exposure prophylaxis (military)	UK approved
LAAV	Kirov Institute/Russia	Live-attenuated (STI-1)	Post-exposure prophylaxis	Approved for use in Russia
GC1109	GC Biopharma (formerly Green Cross)/South Korea	Recombinant rPA	Post-exposure prophylaxis	Phase 1/2 completed; approval under review

### 4.2 Smallpox vaccines

Smallpox is an acute febrile exanthematous disease caused by *Variola virus*, commonly referred to as variola or smallpox. Smallpox vaccines are live-attenuated formulations primarily based on *Vaccinia* virus, a member of the *Orthopoxvirus* genus within the *Poxviridae* family. These vaccines induce robust humoral and cellular immune responses, supporting long-lasting adaptive immunity (24). As a result of widespread vaccination campaigns, the global incidence of smallpox declined dramatically, leading the WHO to declare its eradication in 1980. While routine immunization programs were discontinued in most countries, small-scale vaccination continues for research and biodefense preparedness. Research is actively ongoing to improve the safety profiles of these vaccines and to minimize adverse effects.

Currently, two smallpox vaccines are approved by the U. S. FDA: ACAM2000^®^, manufactured by Emergent BioSolutions, and JYNNEOS^®^, developed by Bavarian Nordic. Previously, the U. S. Department of Defense (DoD) used Dryvax^®^, developed by Wyeth Laboratories (later acquired by Pfizer), for immunizing military personnel. ACAM2000^®^ replaced Dryvax^®^ following FDA approval in 2007. ACAM2000^®^ is derived from a replicating vaccinia virus, while JYNNEOS^®^ is based on a non-replicating strain and was approved in 2019. JYNNEOS^®^ was also used during the 2022 mpox outbreak in Europe and North America.

Smallpox vaccines are classified into four generations based on attenuation methods, production platforms, and safety profiles. First-generation vaccines, such as Dryvax^®^ and Lancy-Vaxinia Berna (Switzerland), were produced using calf lymph, which posed contamination risks from bacteria and other viruses. This led to the development of second-generation vaccines, manufactured using aseptic cell culture systems. ACAM1000^™^ (derived from MRC-5 cells) and ACAM2000^™^ (derived from Vero cells), both developed by Acambis, are representative second-generation examples. ACAM2000^®^ is still stockpiled by the U. S. government and administered to military personnel. CJ-50300, developed by HK inno. N (South Korea), and Elstree-BN, developed by Bavarian Nordic, are also second-generation vaccines. CJ-50300, derived using MRC-5 cells, has completed Phase 3 clinical trials (NCT01056770, NCT01317238) and is being developed as a contact-dispensing coated microneedle (CD MN) patch. Elstree-BN is produced using cell culture techniques and has been stockpiled for emergency use in several European countries.

Third-generation vaccines are based on naturally attenuated strains of *Vaccinia* virus developed through serial passaging to reduce virulence while maintaining immunogenicity. Notable examples include JYNNEOS^®^, derived from Modified Vaccinia Ankara (MVA), and LC16m8 (Lister Clone 16 m8), a variant of the Lister strain developed by KM Biologics in Japan. These strains are propagated using the bacterial artificial chromosome (BAC) system and maintained as plasmid DNA in *Escherichia coli*. LC16m8 is currently licensed and distributed domestically by Kaketsuken in Japan.

Fourth-generation vaccines are genetically engineered live-attenuated vaccines with further reduced virulence. These include NYVAC (New York Vaccinia virus) and NTV (Non-replication Tian Tan strain) (25, 26). Both vaccines have demonstrated favorable immunogenicity and safety profiles (27). The four-generation classification of smallpox vaccines reflects the evolutionary progression in vaccine strain attenuation, production systems, and overall safety characteristics. Representative examples of each generation are summarized in Table 4.

**Table 4:** Representative smallpox vaccines categorized by generation.

Generation	Vaccine name	Developer/country	Platform	Replicating	Status/notes
1st	Dryvax^®^	Wyeth laboratories/United States	Live-attenuated (vaccinia virus) (calf lymph)	Yes	Discontinued; used in early U. S. military
2nd	ACAM2000^™^	Acambis/UK	Live-attenuated (vaccinia virus) (Vero cells)	Yes	FDA approved; in U. S. strategic stockpile
	CJ-50300	HK inno. N/South Korea	Live-attenuated (vaccinia virus) (MRC-5 cells)	Yes	Phase 3 completed in Korea; patch version under development
	Elstree-BN	Bavarian Nordic/Denmark	Live-attenuated (vaccinia virus)	Yes	Strategic stockpile in European countries
3rd	JYNNEOS^®^ (MVA-BN)	Bavarian Nordic/Denmark	Modified vaccinia ankara	No	FDA/EMA approved; also for monkeypox
	LC16m8	KM biologics/Japan	Lister strain	No	Approved in Japan; produced by Kaketsuken
4th	NYVAC	Sanofi/France	Recombinant vaccinia virus	No	Preclinical (experimental)
	NTV	China CDC/China	Non-replicating Tian Tan strain	No	Preclinical (experimental)

Beyond live-attenuated platforms, smallpox vaccine development has expanded to include protein subunit, DNA, and RNA-based approaches. For example, a subunit vaccine containing the L1 protein from the mature virion (MV) membrane has demonstrated antibody induction in mouse models (28). Research into neutralizing peptide candidates targeting *Variola virus* antigens has also shown potential for improving vaccine safety profiles (29).

**Figure 3:**
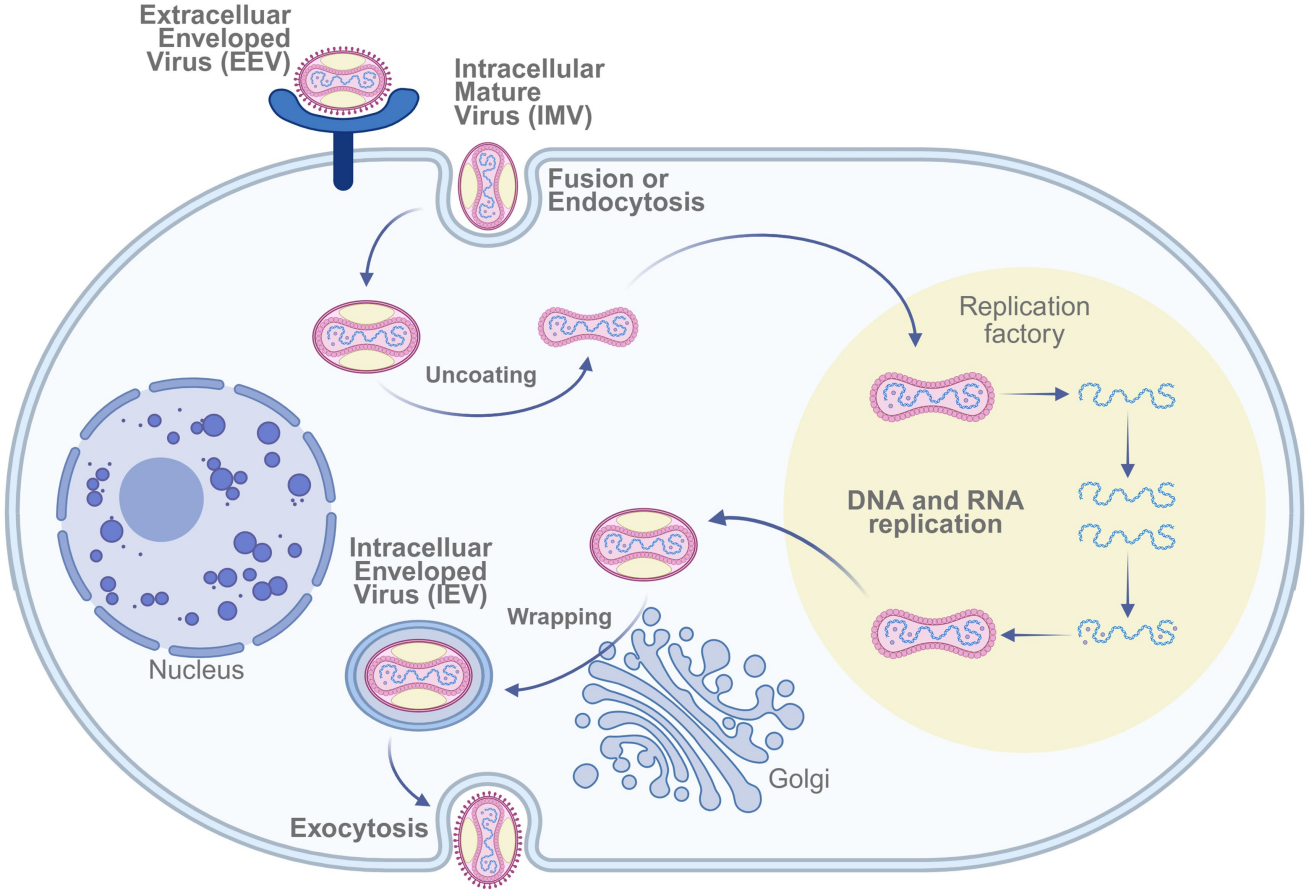
Mechanism of smallpox virus infecting host cells. The replication cycle of Vaccinia virus involves entry via fusion or endocytosis of the intracellular mature virus (IMV), followed by uncoating, DNA/RNA replication within viral factories, wrapping in the Golgi apparatus, and release as extracellular enveloped virus (EEV) through exocytosis. Created in BioRender. https://BioRen-der.com/s5e8sye.

The U. S. Army Medical Research Institute of Infectious Diseases (USAMRIID) developed a DNA vaccine targeting genes expressed by both external enveloped virions (EV) and intracellular mature virions (MV) of the *Variola virus*. This vaccine candidate demonstrated protective efficacy in nonhuman primate models (30). The replication cycle of the *Variola virus*—encompassing both intracellular and extracellular infectious forms—is illustrated in Figure 3.

More recently, mRNA-based vaccines targeting smallpox and mpox have attracted increasing attention. Moderna developed mRNA-1769, a LNP vaccine encoding the *Orthopoxvirus* antigens M1 and A29. This vaccine demonstrated protection in nonhuman primates and is currently being prepared for a Phase 1/2 clinical trial (NCT05995275) (31). *In silico*-designed multi-epitope mRNA vaccines targeting conserved regions across *Variola virus*, *Monkeypox* virus, and *Vaccinia* virus have also been proposed (32).

Additionally, a quadrivalent mRNA vaccine (LAB-LNP) expressing the A27, L1, A33, and B5 antigens of Vaccinia virus showed strong immunogenicity in rodent models (33). Bivalent formulations such as LBA (B6R-A29L) and LAM (A35R-M1R), and a tetravalent formulation LBAAM (B6R-A35R-A29L-M1R), have demonstrated robust immune responses and protective efficacy in murine studies (34).

### 4.3 Plague vaccines

Plague is a systemic infectious disease caused by *Y. pestis*, historically known as the “Black Death.” Clinically, it is classified into bubonic, septicemic, and pneumonic forms. Due to its low infectious dose, transmissibility (via close contact and by vector), and high case fatality rate, *Y. pestis* is designated as a high-priority biological threat.

The earliest vaccine developed was Haffkine’s heat-killed whole-cell (KWC) vaccine, produced from heat-inactivated *Y. pestis*. Although it was employed in India to suppress outbreaks, the vaccine’s limited duration of protection, adverse effects, and lack of efficacy against pneumonic plague hindered widespread adoption. The first plague vaccine approved by the U. S. FDA was Cutter Biological’s Plague Vaccine, USP, manufactured using formalin-inactivated *Y. pestis* strain 195/P. While effective against bubonic plague, it failed to confer adequate protection against the pneumonic form and was discontinued in 1999 due to side effects and the burden of multi-dose administration. To date, no plague vaccine has received FDA approval.

Live-attenuated vaccines include those based on *Y. pestis* EV76 and its variant EV76 NIIEG. These have demonstrated protection against bubonic plague and partial efficacy against pneumonic plague. However, due to reports of adverse events, they are not commercially available and are used only in select endemic regions, such as Russia and China (35).

Advances in genetic engineering and protein purification technologies have led to the development of recombinant protein subunit vaccines targeting key antigens of *Y. pestis*: the capsular protein F1 and the low-calcium response protein LcrV (36). RypVax, developed by PharmAthene, completed Phase 1 trials (NCT00097396, NCT00246467), while DynPort Vaccine Company’s rF1-V fusion vaccine advanced to Phase 2 (NCT00332956, NCT01122784). rV10, developed by Schneewind’s team, is currently under FDA Investigational New Drug (IND) review. However, these vaccines have not demonstrated complete protection against pneumonic plague in nonhuman primate models.

To enhance efficacy, novel adjuvant strategies have been explored. The National Institute of Allergy and Infectious Diseases (NIAID) developed a Flagellin/F1/V fusion vaccine, which completed a Phase 1 trial (NCT01381744) (37). Another subunit vaccine developed by the Jiangsu Centers for Disease Control and Prevention (Jiangsu CDC), utilizing a *Yersinia pseudotuberculosis*-derived F1-rV construct, showed robust protection in mouse models of pneumonic plague when combined with monophosphoryl lipid A (MPLA) adjuvant. This candidate is currently in Phase 2b clinical trial (NCT05330624) (38, 39). Dynavax Technologies also completed a Phase 2 trial of its rF1V vaccine for pneumonic plague (NCT05506969).

Recent developments in plague vaccine research span multiple platforms, including protein subunit, viral vector, DNA, and mRNA-based approaches. Several candidates remain in preclinical stages. A monovalent adenovirus type 5-based recombinant vaccine (rAd5-LcrV) has been developed, and a trivalent formulation (rAd5-YFV), encoding F1, LcrV, and YsF (needle protein) antigens, demonstrated protective efficacy in murine models (40). When co-administered with a recombinant YFV fusion protein via intramuscular injection, the trivalent vaccine also conferred protection in nonhuman primates (41). More recently, researchers at the University of Oxford initiated a Phase 1 clinical trial of a novel chimpanzee adenovirus-based vaccine candidate, which was approved by the European Medicines Agency (EMA).

DNA vaccine candidates encoding F1 and LcrV antigens have shown promising protective effects in murine models. However, co-administration with LcrV protein subunit vaccines has been shown to enhance antibody titers beyond those achieved with DNA vaccination alone (42).

mRNA-based plague vaccines have also gained attention. One candidate encoding the *caf1* gene (F1 antigen) was optimized by modifying guanine (G) and cytosine (C) content and conjugated with human Fc (fragment crystallizable) domains to enhance immunogenicity. This mRNA-LNP vaccine induced high antibody titers in mouse models of bubonic plague and achieved a 50% survival rate in mice exposed to a lethal dose of *Y. pestis* (43).

**Figure 4:**
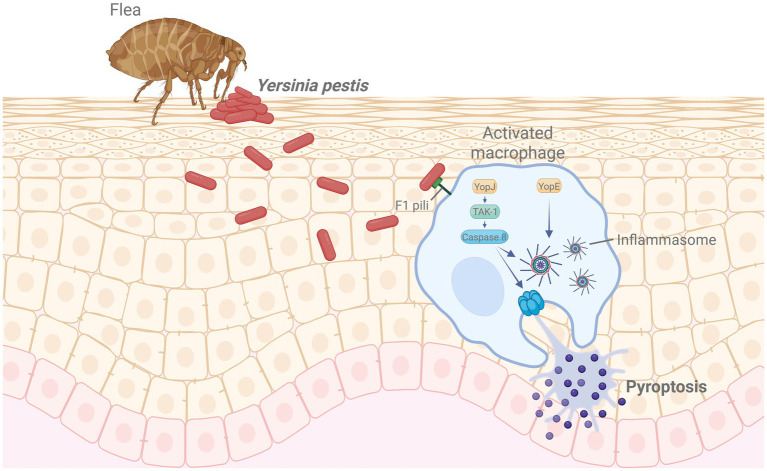
Mechanism of *Yersinia pestis* infection and immune evasion. Following flea-borne transmission, *Y. pestis* invades macrophages using F1 pili and delivers effector proteins such as YopJ and YopE. YopJ inhibits TAK1 signaling, activating caspase-8 and inducing pyroptosis, while YopE facilitates inflammasome activation, promoting immune evasion. Created in BioRender. https://Bio-Render.com/4ohol2n.

Table 5 summarizes representative plague vaccines categorized by platform type, clinical development stage, and targeted disease forms. Figure 4 illustrates the infection mechanism of *Y. pestis* and its immune evasion strategies within host macrophages.

**Table 5:** Representative plague vaccines under development.

Platform	Vaccine example	Target disease form	Clinical stage/status
Inactivated	Plague vaccine, USP	Bubonic plague	Discontinued (1999)
Live-attenuated	EV76, EV76NIIEG	Bubonic and pneumonic plague (partial)	Limited use (Russia, China)
Subunit	RypVax, rF1-V	Bubonic plague	Phase 1/2 completed
Subunit (with adjuvant)	Flagellin/F1/V	Pneumonic plague	Phase 1 completed
	OMV-based F1-rV	Pneumonic plague	Phase 2b ongoing
	Dynavax, rF1V	Pneumonic plague	Phase 2 completed
Viral vector	rAd5-LcrV, rAd5-YFV	Bubonic and pneumonic plague	Preclinical/Phase 1 (UK)
DNA	Plasmid DNA vaccine (F1 and LcrV)	Bubonic plague	Preclinical
mRNA	mRNA-LNP vaccine (*caf1* gene-based)	Bubonic plague	Preclinical

### 4.4 Cholera vaccines

**Figure 5:**
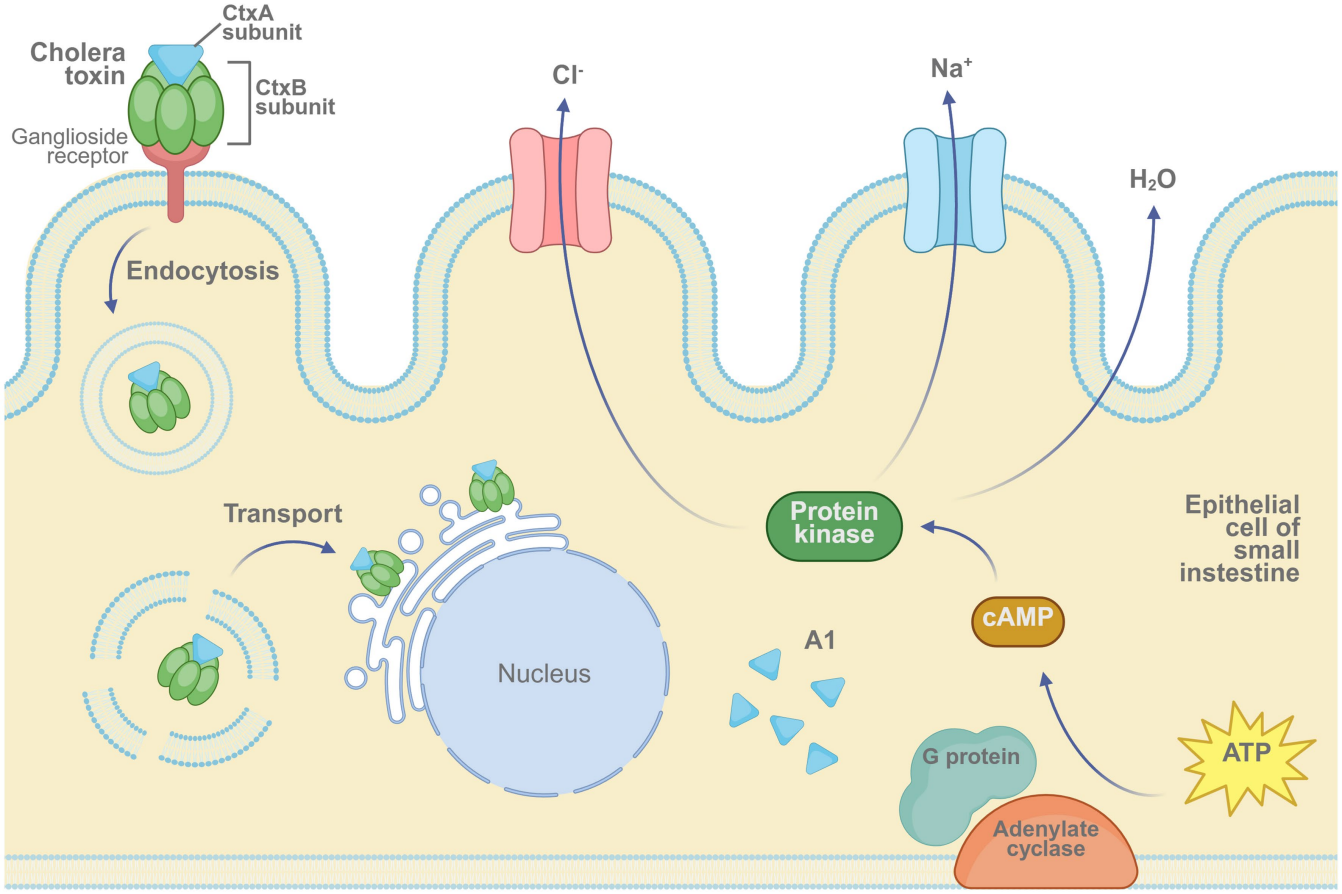
Mechanism of cholera toxin activity in intestinal epithelial cells. The cholera toxin binds to ganglioside receptors via its B subunit and enters the host cell through endocytosis. The A1 fragment of the A subunit activates adenylate cyclase through G protein signaling, leading to an increase in intracellular cAMP levels. This cascade activates protein kinase A (PKA), triggering Cl^−^ and Na^+^ efflux and water secretion, ultimately resulting in severe diarrhea. Created in BioRender. https://BioRender.com/52xqq40.

Cholera is a severe gastrointestinal infection caused by *V. cholerae*, characterized by profuse diarrhea and rapid dehydration, which can lead to high mortality during outbreaks. Figure 5 illustrates the mechanism by which cholera toxin induces ion imbalance and fluid secretion in intestinal epithelial cells. To reduce disease burden, both oral inactivated and live-attenuated vaccines have been developed. Oral formulations are generally preferred over injectable vaccines due to their favorable safety profiles and demonstrated efficacy.

One of the earliest oral cholera vaccines to gain widespread use was the whole cell–recombinant B subunit (WC-rBS) formulation. This vaccine combines inactivated *V. cholerae* O1 serotype strains with a recombinant cholera toxin B subunit (rCtxB) and is marketed as Dukoral^®^ by Valneva SE (France). It received approval from the European Medicines Agency (EMA) for use across the European Union (EU).

Following this, the International Vaccine Institute (IVI) developed a bivalent inactivated whole-cell (bivWC) vaccine that omits rCtxB and includes both O1 and O139 serotypes. The formulation is commercially available as Shanchol^™^, produced by Shantha Biotechnics (India, a Sanofi subsidiary), and Euvichol^®^, manufactured by EuBiologics in South Korea. Euvichol® has since evolved into updated versions—Euvichol-Plus^®^ and Euvichol-S^®^—with Euvichol-S^®^ optimized for improved packaging and lower production costs. Both Shanchol^™^ and Euvichol^®^ are maintained in strategic reserves by Gavi, the Vaccine Alliance, for deployment in large-scale immunization campaigns across cholera-endemic regions (44). In 2023, IVI also initiated clinical trials for DuoChol, a low-cost, capsule-based oral inactivated vaccine. Other domestically approved vaccines include Cholvax by Incepta Vaccine (Bangladesh) and Hillchol^®^ by Bharat Biotech (India).

Among oral live-attenuated vaccines, Vaxchora^®^ (CVD-103-HgR), developed by PaxVax, received FDA approval in 2016. The Advisory Committee on Immunization Practices (ACIP) of the U. S. CDC recommends Vaxchora^®^ for U. S. travelers visiting cholera-endemic areas. It is derived from the O1 Inaba strain, with deletion of the cholera toxin A subunit (CtxA) (45). Another oral live-attenuated candidate, CholeraGarde^®^ by Celldex Therapeutics, has completed Phase 2 clinical trials (NCT00741637).

However, current oral inactivated and live-attenuated vaccines show limited long-term immunity in children under five, likely due to underdeveloped intestinal immune responses (46). To address this challenge, conjugate vaccine strategies have been investigated by linking O-specific polysaccharide (OSP) to carrier proteins. A research team at Harvard Medical School developed an injectable conjugate vaccine by coupling the OSP of the O1 Inaba strain (PIC018) to a recombinant tetanus toxin heavy chain fragment (rTTHc). In mouse models, this vaccine induced strong immune responses and conferred protection (47). The OSP:rTTHc vaccine is currently being prepared for a Phase 1 clinical trial under IVI sponsorship (NCT05559983). In parallel, a Qβ virus-like particle–based conjugate vaccine has also been developed (48).

An emerging vaccine candidate, MucoRice-CTB, is a rice-based oral vaccine expressing the cholera toxin B subunit (CtxB) (49). It has demonstrated immunogenicity in mouse, pig, and nonhuman primate models, and elicited antigen-specific serum IgG and IgA responses in a human Phase 1 trial (Table 6). Additionally, a DNA vaccine encoding ctxB has shown the ability to induce immune responses in mice (oral delivery) and rabbits (injection-based delivery) (50, 51).

**Table 6:** Representative cholera vaccines and their development status.

Platform	Vaccine example	Target population/notes	Clinical stage/status
Inactivated (oral)	Dukoral^®^ (WC-rBS)	General population (EMA approved)	Approved (EU)
	Shanchol^™^, Euvichol^®^, Euvichol-Plus^®^/S^®^ (bivWC)	Used in mass vaccination (Gavi-supported)	Approved (WHO PQ)
	DuoChol	Low-cost capsule format	Preclinical; ready for clinical trial
	Cholvax, Hillchol®	National use (Bangladesh, India)	Approved for national use
Live-attenuated (oral)	Vaxchora (CVD-103-HgR)	US travelers to endemic areas	FDA approved
	CholeraGarde^®^	–	Phase 2 completed
Conjugate (injectable)	OSP:rTTHc (Harvard)	Pediatric focus, enhanced mucosal immunity	Recruiting Phase 1
	OSP-Qβ VLP conjugate	Strong antibody induction	Preclinical
Plant-based (oral)	MucoRice-CTB	Human trial completed (IgG, IgA responses)	Phase 1 completed

### 4.5 Botulism vaccines

**Figure 6:**
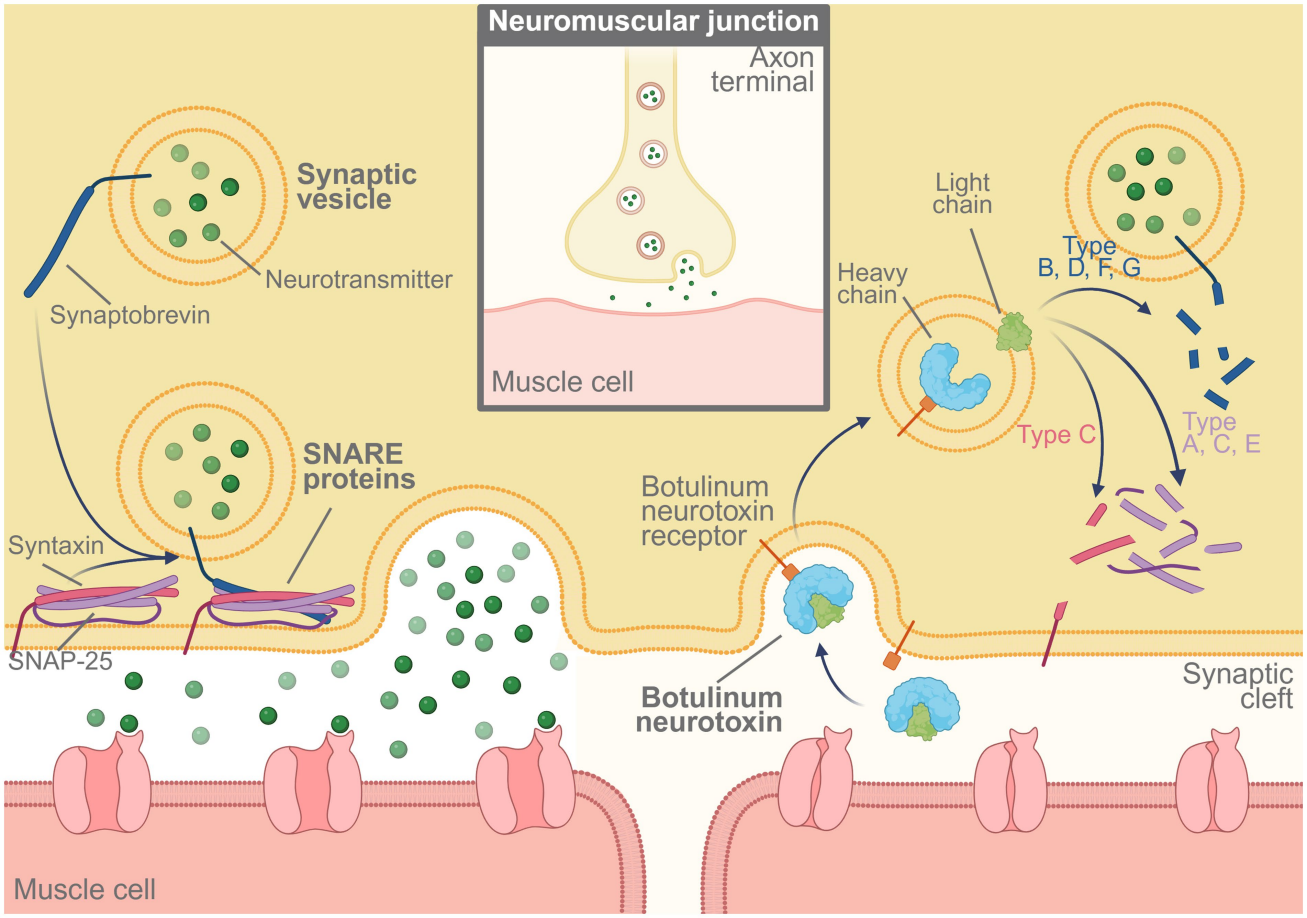
Mechanism of action of botulinum neurotoxin. BoNT binds to its receptor at the neuromuscular junction, enters the presynaptic neuron, and cleaves SNARE proteins (including SNAP-25, syntaxin, and synaptobrevin). This cleavage prevents the release of acetylcholine, resulting in muscle paralysis. Created in BioRender. https://BioRender.com/si2qwsy.

Botulism is a severe neuroparalytic disorder caused by exposure to botulinum neurotoxins (BoNTs), which can induce life-threatening paralysis by inhibiting neuromuscular transmission, even at extremely low doses. BoNTs are classified into eight serotypes (A–H), with types A, B, and E most commonly associated with human infections. Type F has also been reported in rare cases. The mechanism by which BoNTs disrupt neuromuscular function involves serotype-specific cleavage of SNARE (Soluble NSF Attachment Protein Receptor) proteins, thereby preventing acetylcholine release at the neuromuscular junction (see Figure 6). Although there is currently no evidence of antibody-dependent enhancement (ADE) associated with BoNT vaccination, theoretical concerns have been raised regarding potential immunological complications if a vaccinated individual is later exposed to a mismatched serotype.

During World War II, the United States developed and administered a bivalent toxoid vaccine targeting BoNT/A and BoNT/B for military personnel. This was followed by a pentavalent toxoid vaccine covering BoNT/A, B, C, D, and E, which was used under an IND application for military and laboratory personnel beginning in 1965. Due to concerns regarding side effects and reduced efficacy from long-term storage, the vaccine was discontinued in 2011. To date, no botulism vaccine has received full approval from the U. S. FDA. Commercially, Longrange^®^, developed by Zoetis (Australia), is approved for veterinary use against BoNT/C and BoNT/D in cattle.

Recent efforts to advance botulism vaccine development have concentrated on recombinant protein subunit platforms. Multiple candidates have been engineered using heavy chain (Hc) domains of botulinum neurotoxins (BoNTs), expressed in *E. coli* systems (52). Among these, a vaccine candidate targeting the Hc domain of BoNT serotype B demonstrated protective efficacy in mouse models (53). A separate tetravalent formulation—comprising receptor-binding domains from BoNT serotypes A, B, E, and F—also induced robust immunity in preclinical studies (54). In parallel, catalytically inactive BoNT holoproteins (ciBoNT HPs) have shown promise by eliciting protective responses in similar experimental settings (55). Another noteworthy approach involved a mutant variant of BoNT/A1W, engineered to reduce both catalytic activity and receptor-binding affinity. This modified toxin produced strong neutralizing antibody responses in both cellular assays and animal models (56). Additionally, the California Department of Public Health completed a Phase 2b trial of rBV, a recombinant vaccine designed to protect against infant botulism, targeting individuals with prior immunization via the pentavalent toxoid. The study confirmed elevated neutralizing antibody titers against BoNT/A and BoNT/B (NCT01701999) (57).

Viral vector–based platforms are also being explored. One candidate utilizing an influenza viral vector to express the BoNT/A Hc domain provided protection via intranasal delivery in mice (58). Another vaccine, based on a replication-deficient human adenovirus type 5 vector expressing the BoNT/C Hc C-terminal domain, demonstrated complete protection in murine models (59).

DNA vaccine candidates encoding BoNT Hc receptor-binding domains have also been shown to induce strong humoral immune responses. A plasmid-based vaccine targeting BoNT/A conferred protection and induced cross-reactive antibodies against BoNT/E in mouse models (60, 61). A toxoid vaccine targeting botulinum serotypes C and D, co-administered with aluminum hydroxide nanoparticle-based adjuvants, also elicited high antibody titers and robust immune responses in livestock (62).

Representative botulism vaccine candidates, categorized by platform and clinical development stage, are summarized in Table 7.

**Table 7:** Representative botulism vaccine candidates.

Platform	Vaccine example	Serotype covered	Efficacy
Toxoid	Pentavalent toxoid (IND, discontinued)	A, B, C, D, E	Discontinued use
	C/D toxoid with alum nanoparticle	C, D	Livestock
Protein subunit	BoNT/B Hc subunit	B	Mouse
	Tetravalent botulinum vaccine (TBV)	A, B, E, F	Mouse
	Catalytically inactive BoNT holoprotein (ciBoNT HP)	Each of A, C, E, F	Mouse
	M-BoNT/A1W (mutant holoprotein)	A	Mouse
	rBV A/B	A, B	Human (Phase 2b)
Viral vector	Influenza vector expressing BoNT/A Hc	A	Mouse
	Ad5-BoNT/C Hc	C	Mouse
DNA	BoNT/A receptor domain DNA	A	Mouse
	BoNT/E receptor domain DNA	E	Mouse

## 5 Strategic stockpiling and national preparedness

Biological threats represent a complex challenge at the intersection of national defense and public health—especially in the era of modern warfare and recurring outbreaks of infectious diseases. As these risks continue to evolve, the need for proactive and flexible preparedness strategies has become increasingly clear. Among these strategies, maintaining strategic vaccine stockpiles has emerged as a key pillar of biodefense, particularly in addressing high-risk agents with strong potential for weaponization, such as *B. anthracis*, *Variola virus*, and botulinum toxin.

Beyond reducing illness and death during bioterrorism events, vaccine stockpiles also serve a dual purpose: while supporting public health continuity during emergencies, they are especially critical for maintaining military operational readiness in scenarios involving biological weapons deployment, including forward deployment in contested environments.

To meet these goals, countries like the United States, South Korea, and Japan have established stockpiling systems tailored not only to civilian healthcare needs, but also to military threat assessment, including the capacity to immunized troops ahead of high-risk deployments. These frameworks consider essential logistical components—such as selecting priority pathogens, choosing suitable vaccine platforms, ensuring cold-chain infrastructure, and preparing for rapid deployment. Over time, vaccine stockpiling has become a key feature not only in military contingency planning but also in broader civilian public health and emergency response systems.

Table 8 provides an overview of national and international vaccine stockpiling initiatives. While some programs are explicitly designed to counter bioterrorism threats (e.g., anthrax or smallpox), other focus on pandemic preparedness or endemic disease control. This table includes both types to illustrate the broader landscape of vaccine reserve strategies.

**Table 8:** National and global vaccine stockpiling initiatives for biodefense and public health preparedness.

Country/region	Stockpiled vaccines	Target pathogens	Managing agency	Key strategy summary
United States	BioThrax^®^, CYFENDUS^™^ (Anthrax); ACAM2000^®^, JYNNEOS^®^ (Smallpox)	*Bacillus anthracis*, *Variola virus*, botulinum toxin	HHS, Centers for Disease Control and Prevention (CDC), Administration for Strategic Preparedness and Response (ASPR)	SNS program; 12-h push packages; centralized stockpiling and tiered response plans
European Union	Smallpox vaccines, pandemic vaccines, biological threat countermeasures	High-priority biological agents	European Commission, HERA, RescEU	Joint procurement; strategic reserves under RescEU; supply chain resilience for biological threat preparedness
South Korea	BCG, MMR, Tdap, PPSV (expanding list including DTaP, PCV15, hexavalent vaccine)	Routine and pan-demic pathogens; future potential for biodefense	KDCA, Ministry of Health and Welfare, Ministry of National Defense (MND)	2024–2028 mid-term plan; stage-wise expansion; legal basis and cold chain readiness; integration with military biological response requirements
United Kingdom	Smallpox vaccines	High-threat pathogens (smallpox)	NHS, MoD	Civil-military cooperation for wartime vaccine
Japan	Anthrax, smallpox, botulinum toxin vaccines	Bioterrorism-related pathogens	Ministry of Health, Japan Self-Defense Force	Designated stockpiles for high-threat agents; integration with domestic production; aligned with national bioterrorism and military response frameworks
Global	Cholera, yellow fever, smallpox, COVID-19 vaccines	Epidemic-prone diseases	WHO, Gavi, CEPI	Support for LMICs; emergency deployment through COVAX; global coordination mechanisms

The United States maintains the Strategic National Stockpile (SNS), a centralized federal repository for vaccines and medical countermeasures intended to counter biological threats (63). The SNS contains large reserves of medical equipment, pharmaceuticals, and vaccines—including those for anthrax, smallpox, and botulism—designed for rapid deployment during public health crises (63, 64).

Among these, the anthrax vaccine BioThrax^®^ is administered to high-risk populations, including military and selected civilian groups. In addition, next-generation vaccines such as CYFENDUS™ have been acquired (65). Smallpox vaccines ACAM2000^®^ and JYNNEOS^®^ are preserved in substantial quantities for biodefense purposes, despite global eradication of the disease. Vaccines and antitoxins targeting botulinum toxin are also incorporated in the stockpile (65).

The SNS, managed by the U. S. Department of Health and Human Services (HHS), includes a “12-Hour Push Package” system designed to deliver critical medical countermeasures within 12 h of a national emergency (64). To maintain operational readiness, the stockpile is routinely replenished, while detailed inventory levels and storage locations are kept classified for security purposes (63, 64).

The SNS operates in close coordination with state and local governments, supporting scenario-based planning and prioritization protocols. Key criteria for determining stockpile contents include the virulence, transmissibility, and bioweapon potential of pathogens (66). In the event of an emergency, established protocols prioritize vaccination for military personnel and other high-risk groups (66, 67).

In parallel, the EU has implemented its own coordinated vaccine stockpiling strategy to strengthen preparedness for infectious disease outbreaks and biological threats. This approach is supported by several key programs, including the EU Vaccines Strategy, the RescEU initiative under the EU Civil Protection Mechanism, and the Health Emergency Preparedness and Response Authority (HERA) (68). In the aftermath of the COVID-19 pandemic, the EU expanded its joint procurement system and created vaccine reserves accessible across all member states (68).

Through advance purchase agreements (APAs) with pharmaceutical manufacturers, the EU secures and distributes vaccines across member countries under the supervision of the European Commission. In emergencies, vaccine stockpiles and other critical medical resources can be accessed through the RescEU system (68, 69).

HERA also bridges stockpiling with R&D initiatives, covering vaccines for biological threats as well as other medical countermeasures and CBRN protective supplies (69, 70). The Russia–Ukraine conflict in 2022 prompted further expansion of reserves for CBRN-related threats (70).

However, variations in threat assessments among EU nations and the classification of stockpiling data as national security information complicate efforts to develop a fully integrated system (71). Many countries maintain their stockpile data independently, limiting intergovernmental coordination (71).

In response, the EU is working to strengthen its supply chain resilience and strategic independence through expanded joint procurement and shared inventory frameworks based on member solidarity (71).

South Korea has introduced its own national vaccine stockpiling strategy to stabilize supply and enhance outbreak readiness. Since 2023, the Korea Disease Control and Prevention Agency (KDCA) has implemented the National Vaccine Stockpiling Mid- to Long-Term Plan (2024–2028). Of the 24 vaccines in the National Immunization Program (NIP), four—BCG (*Bacillus Calmette–Guérin*, intradermal), MMR (Measles, Mumps, and Rubella), PPSV (Pneumococcal Polysaccharide Vaccine), and Tdap (Tetanus, diphtheria, and acellular pertussis)—have been prioritized for reserve expansion (72).

As of 2024, the average stockpiling coverage for these four vaccines was 27.6%, with plans in place to reach full (100%) coverage. In the initial phase, three vaccines—DTaP (Diphtheria, Tetanus, and acellular Pertussis vaccine), PCV15 (15-valent Pneumococcal Conjugate Vaccine), and a hexavalent combination vaccine—will be added due to their import-dependency or limited suppliers. A second phase will include 10 domestically manufactured vaccines lacking viable alternatives, with additional candidates planned for evaluation after 2034 (72).

In South Korea, the legal basis for vaccine stockpiling was established in 2019 with the introduction of Article 33–2 of the Infectious Disease Control and Prevention Act. This article provided a formal regulatory framework for the long-term procurement and management of national vaccine reserves, allowing manufacturers, importers, and distributors to participate in maintaining stockpiles. The system also includes protocols for rotating inventory, preserving cold-chain integrity, and conducting on-site inspections to ensure safe storage conditions (72).

To promote domestic vaccine self-reliance, the government categorizes vaccines into primary and secondary priority groups. This classification strategy helps align national reserve planning with local production capacity, thereby improving preparedness for future outbreaks (73). While concerns over North Korea’s potential use of biological weapons continue to grow, South Korea’s current stockpiling policy remains largely focused on epidemic response and supply stability. This indicates a potential gap in defense-oriented planning for biological threats, underscoring the need to expand national reserve strategies to include military contingency scenarios.

Other countries, including the United Kingdom and Japan, have also built strategic vaccine reserves. The UK maintains a large stock of smallpox vaccines through a preparedness system led by the National Health Service (NHS) and Ministry of Defence (MoD). Japan has assembled stockpiles for anthrax, smallpox, and botulinum toxin as part of its bioterrorism response strategy while also expanding its domestic vaccine production infrastructure (74, 75).

International organizations—particularly the WHO—have assumed a central role in promoting equitable vaccine access by managing global stockpiles and coordinating emergency distribution systems. The WHO maintains reserve frameworks for several infectious diseases considered high-risk from a public health perspective, including smallpox, yellow fever, and cholera, and has taken the lead on initiatives such as the COVID-19 Vaccines Global Access (COVAX) Facility, which aims to improve vaccine availability for low- and middle-income countries.

Although Gavi’s activities are primarily focused on public health emergencies the organization contributes to global preparedness by stockpiling vaccines for diseases such as cholera and yellow fever in advance, ensuring rapid deployment during naturally occurring outbreaks. In parallel, the Coalition for Epidemic Preparedness Innovations (CEPI) supports these efforts by funding the development of novel vaccine platforms and managing logistics for emergency response. While not specific to biodefense, CEPI is also working to establish a centralized global database to enhance the visibility and coordination of international vaccine reserves—an infrastructure that may offer useful insights for future biological threat preparedness.

Together, these international stakeholders play a vital role in bolstering global health resilience—supporting national stockpile systems while fostering cross-border collaboration to counter biological threats effectively (75, 76).

## 6 International collaboration and policy responses

Global cooperation and the strategic maintenance of vaccine reserves are considered critical components in managing large-scale health emergencies, including both naturally emerging pandemics and intentional biological threats. The COVID-19 crisis revealed deep-seated weaknesses in the global response system—most notably, the rise of vaccine nationalism and significant inequities in distribution. In response, a number of international partnerships and policy efforts emerged to improve fairness and strengthen global preparedness.

Gavi has played a key role in increasing vaccine availability in low-income nations by stockpiling vital vaccines—such as those for yellow fever and cholera—and supporting their rapid deployment during outbreaks. Building on this experience, Gavi helped establish the COVAX Facility to enable more equitable global distribution of COVID-19 vaccines. However, despite its high-reaching objectives, COVAX faced major hurdles, including supply shortages and competition from advance purchase agreements made by individual countries, which ultimately limited its ability to deliver vaccines to many of the populations it aimed to serve (77).

The CEPI has prioritized the integration of next-generation vaccine development with scalable stockpiling and production infrastructure. CEPI also supports multilateral partnerships to strengthen global vaccine supply chains. In parallel, the WHO has developed international frameworks—such as the International Health Regulations (IHR) and the Emergency Response Framework (ERF)—to guide vaccine sharing, emergency deployment, and global coordination. The WHO’s Global Vaccine Action Plan further contributes to standardizing public health emergency responses across nations (75, 78).

The Global Preparedness Monitoring Board (GPMB) has issued critical assessments of the international COVID-19 response, drawing attention to serious deficiencies in vaccine stockpile planning and distribution logistics. In its review, the board identified several key areas requiring urgent reform: enhanced transparency in data sharing, stronger global governance mechanisms, and the development of more resilient infrastructure to support equitable vaccine access. GPMB also emphasized that the current system—heavily influenced by high-income countries—needs structural change to close long-standing gaps in global health preparedness (79).

Moving forward, enhanced international coordination and more unified policy efforts will be vital for developing inclusive and adaptable vaccine reserve systems. Future priorities should include strengthening international legal instruments, expanding collaborative resource-sharing models, and investing in decentralized vaccine manufacturing. In parallel, there is increasing momentum behind reevaluating the advance purchase agreement system and establishing standardized data-sharing protocols and synchronized distribution frameworks—critical steps toward faster, fairer responses in the face of future public health crises.

## 7 Discussion and future directions

This review examined recent progress in vaccine development and strategic reserve planning aimed at enhancing preparedness for biological threats. Particular attention was given to both national-level approaches and international collaboration. The analysis suggests that vaccine stockpiling can play a critical role in enhancing national and global resilience to biological threats—particularly when guided by strategic prioritization, realistic delivery scenarios, and integration with broader response systems. The COVID-19 crisis exposed critical weaknesses in vaccine distribution and equity, emphasizing the need for coordinated and inclusive preparedness strategies across borders.

A persistent barrier to vaccine development in the field of biodefense is the absence of commercial incentives. Unlike therapeutic areas such as oncology or chronic diseases, vaccines targeting biological threat agents offer limited financial returns, resulting in minimal engagement from the private sector. This underscores the importance of strong governmental support through targeted R&D funding, tax incentives, and capacity-building for emergency production. Vaccine stockpiling may represent a strategic, long-term investment that strengthens both public health security and national defense readiness—particularly when applied to high-priority threats and supported by targeted governmental action.

While concerns persist regarding the strategic asymmetry between attackers and defenders in biological warfare—particularly the possibility that adversaries may simply choose biological agents not covered by existing vaccine stockpiles—such asymmetry is not absolute. Not all pathogens are equally viable as bioweapons; *B. anthracis* and *Variola virus*, for instance, remain prioritized due to their lethality, environmental persistence, and historical precedent. Stockpiling vaccines against these agents may limit their tactical utility and act as a deterrent by forcing adversaries to consider less effective alternatives. Furthermore, vaccine stockpiling is only one element within a layered biodefense architecture that includes surveillance, diagnostics, therapeutics, and protective equipment. Advances in next-generation platforms such as mRNA and saRNA now enable more agile vaccine development, helping to reduce the temporal and strategic gap between emerging threats and response capacity.

Although vaccine stockpiling is essential, the question of whether to implement preemptive vaccination—particularly for military personnel—remains complex. All vaccines carry a risk of adverse reactions, and during peacetime, the probability of exposure may not appear to justify large-scale immunization. This was evident in the failure of the U. S. voluntary smallpox vaccination program for first responders after the September 11 attacks (9/11), where the perceived risk of vaccine side effects outweighed the perceived likelihood of a bioterrorism event. However, military operations conducted in suspected biological threat environments present a fundamentally different risk profile. In scenarios where *B. anthracis* contamination is likely or where wide-area decontamination would be impractical, preemptive vaccination of deployed units can be strategically justified. Unlike civilian populations, deployed military units may face elevated and predicable exposure risks, justifying the need for preemptive vaccination under specific operational conditions. The development of newer-generation vaccines with improved safety profiles may also shift the risk–benefit balance in favor of such targeted pre-exposure strategies.

Barriers to international cooperation often arise from intellectual property restrictions, proprietary technologies, and concerns over market advantage. While vaccine sharing mechanisms are primarily designed for public health emergencies, selective international coordination and transparency can still enhance biodefense preparedness—particularly in aligning strategic priorities, improving situational awareness, and identifying technology gaps. Public–private partnerships that promote open innovation and support the global visibility of national vaccine reserves may help bridge preparedness asymmetries. Although ensuring equitable access is a public health imperative, it may also contribute to a more resilient and cooperative international framework for responding to biological threats.

Looking ahead, future efforts should focus on assessing the performance of national stockpile systems, exploring frameworks for shared international reserves, and establishing collaborative governance structures. Integrating next-generation technologies, such as mRNA, self-amplifying RNA, and innovative adjuvants, into both national and regional reserves will be crucial. Additionally, conducting real-world evaluations linking stockpile readiness to actual emergency response outcomes, along with cost-effectiveness studies of public-private partnerships, will provide the insights needed to refine preparedness strategies. Together, these steps will significantly strengthen global preparedness and ensure more coordinated and rapid responses across both military and civilian domains in the event of biological threats.

While public health preparedness remains a central concern, this review emphasizes a complementary and often underexplored dimension: the strategic use of vaccines as military countermeasures against biological weapons. In scenarios involving highly persistent or highly contagious agents—such as *B. anthracis* or *Variola virus*—stockpiled vaccines may serve not only as protective tools but also as instruments of deterrence, dissuading adversaries from employing such agents. Thus, vaccine stockpiling should be viewed as a dual-use strategy that enhances both national defense readiness and civilian health security. Future policy and investment decisions must reflect this dual imperative to ensure effective responses in both military and civilian biodefense context.
